# Epibisde­hydro­neotuberostemonine J

**DOI:** 10.1107/S1600536813021077

**Published:** 2013-08-03

**Authors:** Lu Jin, Rong-Rong Zhang, Hai-Yan Tian, Paul Pui-Hay But, Ren-Wang Jiang

**Affiliations:** a Guangdong Province Key Laboratory of Pharmacodynamic Constituents of Traditional Chinese Medicine and New Drugs Research, Institute of Traditional Chinese Medicine and Natural Products, Jinan University, Guangzhou 510632, People’s Republic of China; b School of Life Sciences, The Chinese University of Hong Kong, Shatin, New Territories, Hong Kong SAR, People’s Republic of China

## Abstract

The title compound, C_22_H_29_NO_4_, a stemona alkaloid, is composed of two lactone rings (*A* and *E*), a six-membered ring (*B*), a pyrrole ring (*C*) and a seven-membered ring (*D*). The five-membered rings *A* and *E* exhibit envelope conformations (C atoms as flaps) while ring *C* is planar. Ring *B* exhibits a twist-chair conformation due to fusion with pyrrole ring *C* while ring *D* adopts a chair conformation. The junction between rings *A* and *B* is *cis*. In the crystal, weak C—H⋯O inter­actions involving the two carbonyl groups, a methyl­ene and a methyl group give rise to a three-dimensional network.

## Related literature

For general background to the structures and biological activity of stemona alkaloids, see: Pilli *et al.* (2010 ▶). For the anti­tussive activity of epibisde­hydro­neotuberostemonine J and other stemona alkaloids, see: Chung *et al.* (2003 ▶); Xu *et al.* (2010 ▶). For other properties of and studies on Stemona alkaloids, see: Chung *et al.* (2003 ▶); Frankowski *et al.* (2008 ▶, 2011 ▶); Jiang *et al.* (2006 ▶); Zhang *et al.* (2011 ▶). For an absolute structure reference, see: Jiang *et al.* (2010 ▶). For related isomers, see: Pham *et al.* (2002 ▶).
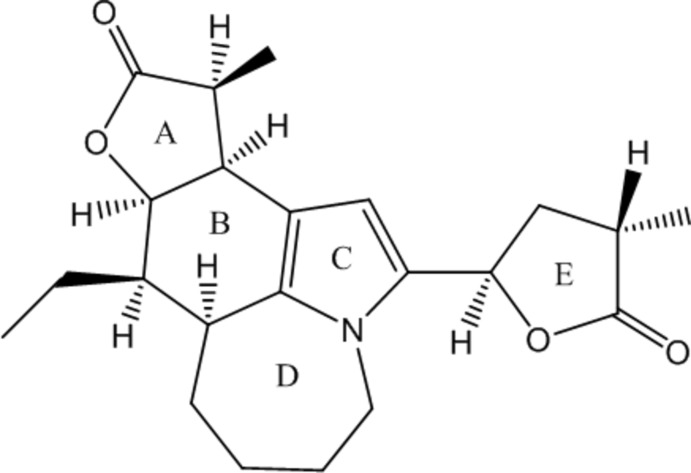


## Experimental

### 

#### Crystal data

C_22_H_29_NO_4_
*M* *_r_* = 371.46
Monoclinic, 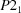

*a* = 6.3596 (19) Å
*b* = 18.495 (3) Å
*c* = 8.3875 (15) Å
β = 92.521 (18)°
*V* = 985.6 (4) Å^3^
*Z* = 2
Mo *K*α radiation
μ = 0.09 mm^−1^
*T* = 291 K
0.43 × 0.28 × 0.20 mm

#### Data collection

Bruker SMART 1000 CCD diffractometer
Absorption correction: multi-scan (*SADABS*; Sheldrick, 2004 ▶) *T*_min_ = 0.831, *T*_max_ = 1.000
2449 measured reflections
1914 independent reflections
1383 reflections with *I* > 2σ(*I*)
*R*_int_ = 0.022

#### Refinement

*R*[*F* ^2^ > 2σ(*F* ^2^)] = 0.045
*wR*(*F* ^2^) = 0.093
*S* = 1.05
1914 reflections
245 parameters
1 restraint
H-atom parameters constrained
Δρ_max_ = 0.13 e Å^−3^
Δρ_min_ = −0.13 e Å^−3^

### 

Data collection: *SMART* (Bruker, 1998 ▶); cell refinement: *SMART* and *SAINT* (Bruker, 1998 ▶); data reduction: *SAINT* and *XPREP* (Bruker, 1998 ▶); program(s) used to solve structure: *SHELXS97* (Sheldrick, 2008 ▶); program(s) used to refine structure: *SHELXL97* (Sheldrick, 2008 ▶); molecular graphics: *XP* in *SHELXTL* (Sheldrick, 2008 ▶); software used to prepare material for publication: *SHELXTL*.

## Supplementary Material

Crystal structure: contains datablock(s) I, global. DOI: 10.1107/S1600536813021077/zl2558sup1.cif

Structure factors: contains datablock(s) I. DOI: 10.1107/S1600536813021077/zl2558Isup2.hkl

Supplementary material file. DOI: 10.1107/S1600536813021077/zl2558Isup3.cml

Additional supplementary materials:  crystallographic information; 3D view; checkCIF report

## Figures and Tables

**Table 1:** Hydrogen-bond geometry (Å, °)

*D*—H⋯*A*	*D*—H	H⋯*A*	*D*⋯*A*	*D*—H⋯*A*
C5—H5*A*⋯O2^i^	0.97	2.60	3.531 (4)	161
C5—H5*B*⋯O4^ii^	0.97	2.66	3.595 (3)	162
C22—H22*B*⋯O4^iii^	0.96	2.63	3.496 (4)	150

Symmetry codes: (i) 
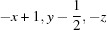
; (ii) 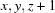; (iii) 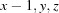.

**Fig. 1.**
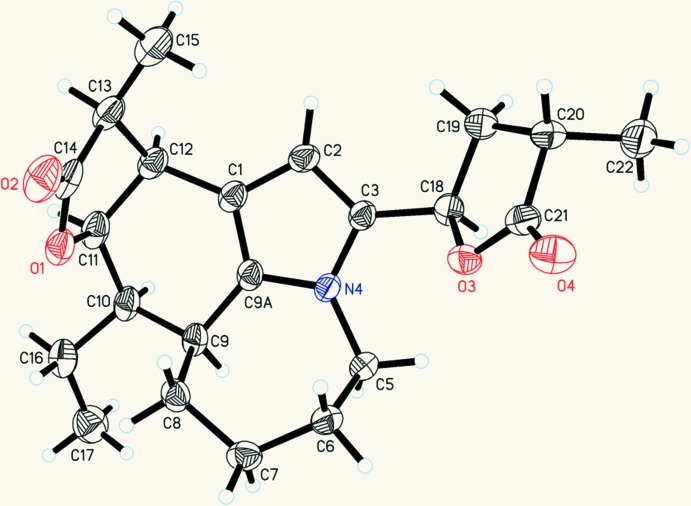
The molecular structure of the title compound showing 30% probability displacement ellipsoids and the atom-numbering scheme.

**Fig. 2.**
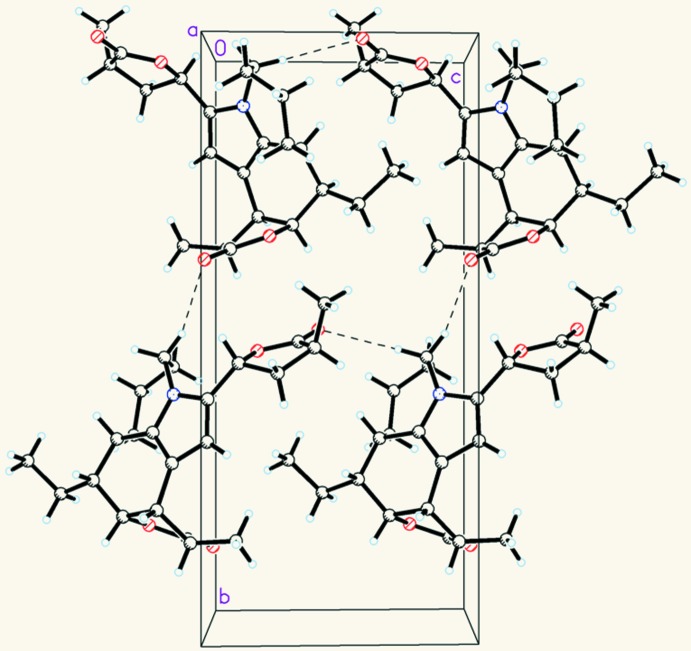
Packing diagram viewed down the a axis.

## supplementary crystallographic information

### 1. Comment

Radix Stemonae extracts derived from the root of Stemona tuberosa (Stemonaceae family) are often used as an antitussive drug to treat respiratory disorders. The alkaloids were found to be the major components responsible for the antitussive activity (Xu *et al.*, 2010). The intriguing structures and pharmacological activities of this fascinating class of compounds have attracted considerable attention (Pilli *et al.*, 2010), and a number of total syntheses (Frankowski *et al.*, 2008), structural modifications (Frankowski *et al.*, 2011) and phytochemical studies (Jiang *et al.*, 2006, Zhang *et al.*, 2011) on new Stemona alkaloids have appeared in recent years.

The title compound C_22_H_29_N_1_O_4_ (Fig. 1) is a Stemona alkaloid. It was first isolated from the roots of Stemona tuberosa ten years ago (Chung *et al.*, 2003) and found to show antitussive activity (Chung *et al.*, 2003); however, its crystal structure had not been reported.

During our on-going search for antitussive natural products, epibisdehydroneotuberostemonine J was isolated again from Stemona tuberosa. It is an isomer of bisdehydroneotuberostemonine (Pham *et al.*, 2002) at C-9 and C-18. The molecule is composed of two lactone ring (A and E), a six-membered ring (B), a pyrrole ring (C) and a seven-membered ring (D). The five-membered rings A and E exhibit envelope conformations while ring C is planar. The six-membered ring B exhibits a twist chair conformation due to fusion with the pyrrole ring C. The seven-membered ring D adopts a chair conformation, in which the atoms C-5, C-6, C-8, C-9 form a plane with a mean deviation of 0.043 (2) Å, and the atoms C-9 A, N-4 and C-7 displaced by -1.070 (3), -1.040 (2) and 0.662 (4) Å from the plane, respectively.

Weak intermolecular C–H···O interactions (Table 1) involving the two carbonyl groups (O-2 and O-4), a methylene (C-5) and a methyl group (C-22) give a three-dimensional structure.

### 2. Experimental

A dry ground herbal sample of Radix Stemonae (5.0 kg) was suspended in 95% EtOH (10 *L*) and heated for two hours to reflux of the solvent. After filtration, the solvent was evaporated under reduced pressure. The residue was acidified with 4% HCl (400 ml) and filtered with Whatman filter papers, then the filtrate (acidic aqueous solution) was washed with diethyl ether (500 ml). The H_2_O layer was basified to pH = 9 with aqueous ammonia (35%) and then extracted with Et_2_O (500 ml). The Et_2_O layer was evaporated to afford the crude alkaloids (15 g), which were subjected to column chromatography over silica gel, and eluted with chloroform: methanol: amonia (98: 2: 0.05) to yield ten fractions. Fraction 3 (2 g), a low polar fraction with an Rf value larger than 0.7 on a normal phase TLC plate (mobile phase cyclohexane: ethyl acetate 1: 1), was subjected to a second separation by silica-gel chromatography with cyclohexane: ethyl acetate (7: 3) as the eluent to yield the title compound (180 mg, colorless powder, Rf = 0.76 at the same TLC condition as bulk fraction 3), which was identified by comparision of the physical and spectroscopic data with the literature (Chung *et al.*, 2003). Colorless crystals suitable for single crystal diffraction were obtained from a mixture of cyclohexane: ethyl acetate at room temperature.

### 3. Refinement

The C-bound H atoms were positioned geometrically and were included in the refinement in the riding-model approximation, with C—H = 0.96 Å (CH_3_) and *U*_iso_(H) = 1.5*U*_eq_(C); 0.97 Å (CH_2_) and *U*_iso_(H) = 1.2*U*_eq_(C); 0.98 Å (CH) and *U*_iso_(H) = 1.2*U*_eq_(C). In the absence of anomalous scatterers and a low Friedel pair coverage the absolute configuration was assigned based on the closely related reference molecule neostenine with known configurations at C-10 and C-13 (Jiang *et al.* (2010)). The highest residual electron density is 0.13 and of no physical meaning.

### Crystal data

**Table:**

C_22_H_29_NO_4_	*F*(000) = 400
*M**_r_* = 371.46	*D*_x_ = 1.252 Mg m^−^^3^
Monoclinic, *P*2_1_	Mo *K*α radiation, λ = 0.71073 Å
*a* = 6.3596 (19) Å	Cell parameters from 2449 reflections
*b* = 18.495 (3) Å	θ = 2.2–24.0°
*c* = 8.3875 (15) Å	µ = 0.09 mm^−^^1^
β = 92.521 (18)°	*T* = 291 K
*V* = 985.6 (4) Å^3^	Prism, colorless
*Z* = 2	0.43 × 0.28 × 0.20 mm

### Data collection

**Table:**

Bruker SMART 1000 CCD diffractometer	1383 reflections with *I* > 2σ(*I*)
Radiation source: sealed tube	*R*_int_ = 0.022
ω scan	θ_max_ = 25.0°, θ_min_ = 2.2°
Absorption correction: multi-scan (*SADABS*; Sheldrick, 2004)	*h* = −1→7
*T*_min_ = 0.831, *T*_max_ = 1.000	*k* = −1→21
2449 measured reflections	*l* = −9→9
1914 independent reflections	

### Refinement

**Table:**

Refinement on *F*^2^	Secondary atom site location: difference Fourier map
Least-squares matrix: full	Hydrogen site location: inferred from neighbouring sites
*R*[*F*^2^ > 2σ(*F*^2^)] = 0.045	H-atom parameters constrained
*wR*(*F*^2^) = 0.093	*w* = 1/[σ^2^(*F*_o_^2^) + (0.0368*P*)^2^ + 0.0285*P*] where *P* = (*F*_o_^2^ + 2*F*_c_^2^)/3
*S* = 1.05	(Δ/σ)_max_ < 0.001
1914 reflections	Δρ_max_ = 0.13 e Å^−^^3^
245 parameters	Δρ_min_ = −0.13 e Å^−^^3^
1 restraint	Extinction correction: *SHELXL97* (Sheldrick, 2008), Fc^*^=kFc[1+0.001xFc^2^λ^3^/sin(2θ)]^-1/4^
Primary atom site location: structure-invariant direct methods	Extinction coefficient: 0.013 (2)

### Special details

**Table:**

Geometry. All e.s.d.'s (except the e.s.d. in the dihedral angle between two l.s. planes) are estimated using the full covariance matrix. The cell e.s.d.'s are taken into account individually in the estimation of e.s.d.'s in distances, angles and torsion angles; correlations between e.s.d.'s in cell parameters are only used when they are defined by crystal symmetry. An approximate (isotropic) treatment of cell e.s.d.'s is used for estimating e.s.d.'s involving l.s. planes.
Refinement. Refinement of *F*^2^ against ALL reflections. The weighted *R*-factor *wR* and goodness of fit *S* are based on *F*^2^, conventional *R*-factors *R* are based on *F*, with *F* set to zero for negative *F*^2^. The threshold expression of *F*^2^ > σ(*F*^2^) is used only for calculating *R*-factors(gt) *etc*. and is not relevant to the choice of reflections for refinement. *R*-factors based on *F*^2^ are statistically about twice as large as those based on *F*, and *R*- factors based on ALL data will be even larger.

### Fractional atomic coordinates and isotropic or equivalent isotropic displacement parameters (Å^2^)

**Table:**

	*x*	*y*	*z*	*U*_iso_*/*U*_eq_	
O1	0.5763 (4)	0.32620 (15)	0.2326 (4)	0.0552 (8)	
O2	0.6531 (5)	0.36723 (17)	−0.0073 (4)	0.0716 (9)	
O3	0.4293 (4)	0.02284 (15)	−0.1869 (3)	0.0472 (7)	
O4	0.5576 (4)	−0.01361 (17)	−0.4150 (3)	0.0611 (9)	
C1	0.2603 (6)	0.2093 (2)	0.1225 (5)	0.0451 (11)	
C2	0.1727 (6)	0.1714 (2)	−0.0118 (5)	0.0469 (10)	
H2A	0.0781	0.1903	−0.0886	0.056*	
C3	0.2514 (6)	0.1021 (2)	−0.0095 (5)	0.0418 (10)	
N4	0.3821 (5)	0.09614 (16)	0.1254 (4)	0.0404 (8)	
C5	0.5164 (6)	0.0346 (2)	0.1696 (5)	0.0449 (10)	
H5A	0.4792	−0.0063	0.1015	0.054*	
H5B	0.4929	0.0208	0.2790	0.054*	
C6	0.7464 (6)	0.0528 (2)	0.1536 (5)	0.0483 (11)	
H6A	0.8247	0.0079	0.1468	0.058*	
H6B	0.7611	0.0785	0.0540	0.058*	
C7	0.8456 (6)	0.0977 (2)	0.2873 (5)	0.0505 (11)	
H7A	0.9952	0.1014	0.2707	0.061*	
H7B	0.8298	0.0720	0.3868	0.061*	
C8	0.7586 (6)	0.1742 (2)	0.3066 (5)	0.0472 (10)	
H8A	0.7729	0.2005	0.2076	0.057*	
H8B	0.8419	0.1991	0.3892	0.057*	
C9A	0.3875 (6)	0.1609 (2)	0.2037 (5)	0.0426 (10)	
C9	0.5259 (6)	0.1751 (2)	0.3501 (4)	0.0440 (10)	
H9A	0.5059	0.1348	0.4236	0.053*	
C10	0.4468 (7)	0.2435 (2)	0.4320 (5)	0.0501 (11)	
H10A	0.3150	0.2295	0.4798	0.060*	
C11	0.3881 (7)	0.3036 (2)	0.3145 (5)	0.0557 (12)	
H11A	0.3360	0.3449	0.3742	0.067*	
C12	0.2296 (6)	0.2868 (2)	0.1766 (5)	0.0493 (11)	
H12A	0.0855	0.2939	0.2102	0.059*	
C13	0.2864 (7)	0.3445 (2)	0.0563 (5)	0.0561 (12)	
H13A	0.2310	0.3903	0.0954	0.067*	
C14	0.5207 (8)	0.3479 (2)	0.0821 (6)	0.0556 (12)	
C15	0.2115 (7)	0.3382 (3)	−0.1162 (6)	0.0708 (14)	
H15A	0.2599	0.3792	−0.1745	0.106*	
H15B	0.0605	0.3368	−0.1232	0.106*	
H15C	0.2665	0.2947	−0.1608	0.106*	
C16	0.5885 (7)	0.2711 (3)	0.5694 (5)	0.0615 (13)	
H16A	0.5235	0.3133	0.6154	0.074*	
H16B	0.7215	0.2863	0.5279	0.074*	
C17	0.6320 (8)	0.2152 (3)	0.7012 (6)	0.0754 (15)	
H17A	0.7226	0.2360	0.7836	0.113*	
H17B	0.6990	0.1736	0.6573	0.113*	
H17C	0.5016	0.2009	0.7454	0.113*	
C18	0.2231 (5)	0.0417 (2)	−0.1246 (5)	0.0438 (10)	
H18A	0.1681	−0.0004	−0.0688	0.053*	
C19	0.0829 (7)	0.0565 (2)	−0.2721 (5)	0.0574 (12)	
H19A	−0.0591	0.0393	−0.2578	0.069*	
H19B	0.0778	0.1078	−0.2957	0.069*	
C20	0.1851 (6)	0.0151 (3)	−0.4045 (5)	0.0504 (11)	
H20A	0.1825	0.0449	−0.5011	0.060*	
C21	0.4088 (6)	0.0065 (2)	−0.3421 (5)	0.0457 (10)	
C22	0.0893 (7)	−0.0577 (3)	−0.4437 (7)	0.0806 (16)	
H22A	0.1641	−0.0799	−0.5278	0.121*	
H22B	−0.0558	−0.0516	−0.4776	0.121*	
H22C	0.0985	−0.0879	−0.3507	0.121*	

### Atomic displacement parameters (Å^2^)

**Table:**

	*U*^11^	*U*^22^	*U*^33^	*U*^12^	*U*^13^	*U*^23^
O1	0.0521 (19)	0.0380 (17)	0.076 (2)	−0.0075 (15)	0.0086 (17)	0.0000 (16)
O2	0.072 (2)	0.051 (2)	0.093 (2)	−0.0096 (18)	0.0255 (19)	0.0111 (19)
O3	0.0362 (15)	0.0522 (17)	0.0528 (17)	0.0054 (14)	−0.0032 (13)	−0.0032 (16)
O4	0.0454 (18)	0.079 (2)	0.0597 (18)	0.0066 (18)	0.0107 (16)	0.0065 (18)
C1	0.038 (2)	0.036 (2)	0.062 (3)	−0.001 (2)	0.009 (2)	0.002 (2)
C2	0.032 (2)	0.041 (2)	0.068 (3)	0.003 (2)	−0.001 (2)	0.005 (2)
C3	0.034 (2)	0.034 (2)	0.057 (3)	−0.0011 (19)	0.002 (2)	−0.001 (2)
N4	0.0352 (17)	0.0295 (18)	0.056 (2)	0.0002 (16)	0.0012 (16)	−0.0011 (17)
C5	0.047 (2)	0.036 (2)	0.052 (2)	0.005 (2)	−0.001 (2)	0.001 (2)
C6	0.042 (2)	0.042 (2)	0.061 (3)	0.006 (2)	−0.002 (2)	0.000 (2)
C7	0.038 (2)	0.053 (3)	0.060 (3)	0.002 (2)	0.002 (2)	0.008 (2)
C8	0.038 (2)	0.046 (2)	0.058 (3)	−0.008 (2)	0.0031 (19)	−0.004 (2)
C9A	0.035 (2)	0.036 (2)	0.058 (3)	−0.0071 (19)	0.006 (2)	−0.002 (2)
C9	0.044 (2)	0.036 (2)	0.052 (2)	−0.007 (2)	0.0098 (19)	−0.005 (2)
C10	0.048 (3)	0.037 (2)	0.066 (3)	−0.008 (2)	0.013 (2)	−0.004 (2)
C11	0.054 (3)	0.040 (3)	0.074 (3)	0.002 (2)	0.018 (3)	−0.015 (2)
C12	0.041 (2)	0.038 (2)	0.070 (3)	0.004 (2)	0.010 (2)	0.001 (2)
C13	0.059 (3)	0.033 (2)	0.076 (3)	0.009 (2)	0.009 (3)	0.002 (2)
C14	0.071 (3)	0.024 (2)	0.074 (3)	−0.004 (2)	0.015 (3)	0.001 (2)
C15	0.074 (3)	0.047 (3)	0.092 (4)	0.006 (3)	0.009 (3)	0.008 (3)
C16	0.072 (3)	0.053 (3)	0.061 (3)	−0.009 (3)	0.010 (3)	−0.012 (3)
C17	0.083 (4)	0.077 (4)	0.066 (3)	0.000 (3)	0.003 (3)	−0.008 (3)
C18	0.029 (2)	0.044 (3)	0.059 (2)	−0.001 (2)	0.0064 (19)	−0.006 (2)
C19	0.042 (2)	0.054 (3)	0.076 (3)	0.008 (2)	−0.012 (2)	−0.008 (3)
C20	0.045 (2)	0.051 (3)	0.055 (2)	0.008 (2)	−0.008 (2)	0.004 (2)
C21	0.042 (2)	0.043 (2)	0.052 (3)	0.002 (2)	−0.001 (2)	0.007 (2)
C22	0.055 (3)	0.062 (3)	0.123 (4)	0.008 (3)	−0.020 (3)	−0.022 (3)

### Geometric parameters (Å, º)

**Table:**

O1—C14	1.356 (5)	C10—C16	1.519 (5)
O1—C11	1.467 (5)	C10—C11	1.520 (6)
O2—C14	1.207 (5)	C10—H10A	0.9800
O3—C21	1.337 (4)	C11—C12	1.533 (6)
O3—C18	1.475 (4)	C11—H11A	0.9800
O4—C21	1.207 (4)	C12—C13	1.523 (6)
C1—C9A	1.368 (5)	C12—H12A	0.9800
C1—C2	1.420 (5)	C13—C14	1.498 (6)
C1—C12	1.518 (6)	C13—C15	1.507 (6)
C2—C3	1.375 (5)	C13—H13A	0.9800
C2—H2A	0.9300	C15—H15A	0.9600
C3—N4	1.378 (5)	C15—H15B	0.9600
C3—C18	1.483 (5)	C15—H15C	0.9600
N4—C9A	1.366 (5)	C16—C17	1.530 (6)
N4—C5	1.462 (5)	C16—H16A	0.9700
C5—C6	1.513 (5)	C16—H16B	0.9700
C5—H5A	0.9700	C17—H17A	0.9600
C5—H5B	0.9700	C17—H17B	0.9600
C6—C7	1.512 (5)	C17—H17C	0.9600
C6—H6A	0.9700	C18—C19	1.518 (5)
C6—H6B	0.9700	C18—H18A	0.9800
C7—C8	1.529 (6)	C19—C20	1.518 (6)
C7—H7A	0.9700	C19—H19A	0.9700
C7—H7B	0.9700	C19—H19B	0.9700
C8—C9	1.540 (5)	C20—C21	1.503 (5)
C8—H8A	0.9700	C20—C22	1.508 (6)
C8—H8B	0.9700	C20—H20A	0.9800
C9A—C9	1.502 (5)	C22—H22A	0.9600
C9—C10	1.534 (6)	C22—H22B	0.9600
C9—H9A	0.9800	C22—H22C	0.9600
			
C14—O1—C11	109.6 (3)	C1—C12—C11	109.1 (3)
C21—O3—C18	110.4 (3)	C13—C12—C11	101.0 (3)
C9A—C1—C2	106.0 (3)	C1—C12—H12A	110.3
C9A—C1—C12	123.3 (4)	C13—C12—H12A	110.3
C2—C1—C12	130.7 (4)	C11—C12—H12A	110.3
C3—C2—C1	108.6 (4)	C14—C13—C15	114.4 (4)
C3—C2—H2A	125.7	C14—C13—C12	101.3 (4)
C1—C2—H2A	125.7	C15—C13—C12	120.5 (4)
C2—C3—N4	107.0 (4)	C14—C13—H13A	106.6
C2—C3—C18	131.3 (4)	C15—C13—H13A	106.6
N4—C3—C18	121.7 (3)	C12—C13—H13A	106.6
C9A—N4—C3	109.1 (3)	O2—C14—O1	120.4 (4)
C9A—N4—C5	124.0 (3)	O2—C14—C13	129.8 (5)
C3—N4—C5	126.5 (3)	O1—C14—C13	109.9 (4)
N4—C5—C6	111.1 (3)	C13—C15—H15A	109.5
N4—C5—H5A	109.4	C13—C15—H15B	109.5
C6—C5—H5A	109.4	H15A—C15—H15B	109.5
N4—C5—H5B	109.4	C13—C15—H15C	109.5
C6—C5—H5B	109.4	H15A—C15—H15C	109.5
H5A—C5—H5B	108.0	H15B—C15—H15C	109.5
C7—C6—C5	115.5 (3)	C10—C16—C17	113.8 (4)
C7—C6—H6A	108.4	C10—C16—H16A	108.8
C5—C6—H6A	108.4	C17—C16—H16A	108.8
C7—C6—H6B	108.4	C10—C16—H16B	108.8
C5—C6—H6B	108.4	C17—C16—H16B	108.8
H6A—C6—H6B	107.5	H16A—C16—H16B	107.7
C6—C7—C8	116.5 (3)	C16—C17—H17A	109.5
C6—C7—H7A	108.2	C16—C17—H17B	109.5
C8—C7—H7A	108.2	H17A—C17—H17B	109.5
C6—C7—H7B	108.2	C16—C17—H17C	109.5
C8—C7—H7B	108.2	H17A—C17—H17C	109.5
H7A—C7—H7B	107.3	H17B—C17—H17C	109.5
C7—C8—C9	113.1 (3)	O3—C18—C3	108.9 (3)
C7—C8—H8A	109.0	O3—C18—C19	104.7 (3)
C9—C8—H8A	109.0	C3—C18—C19	116.4 (3)
C7—C8—H8B	109.0	O3—C18—H18A	108.9
C9—C8—H8B	109.0	C3—C18—H18A	108.9
H8A—C8—H8B	107.8	C19—C18—H18A	108.9
N4—C9A—C1	109.4 (3)	C20—C19—C18	104.5 (3)
N4—C9A—C9	123.3 (3)	C20—C19—H19A	110.9
C1—C9A—C9	127.2 (4)	C18—C19—H19A	110.9
C9A—C9—C10	108.6 (3)	C20—C19—H19B	110.9
C9A—C9—C8	109.8 (3)	C18—C19—H19B	110.9
C10—C9—C8	116.9 (3)	H19A—C19—H19B	108.9
C9A—C9—H9A	107.0	C21—C20—C22	110.4 (4)
C10—C9—H9A	107.0	C21—C20—C19	103.2 (3)
C8—C9—H9A	107.0	C22—C20—C19	115.3 (4)
C16—C10—C11	111.5 (3)	C21—C20—H20A	109.2
C16—C10—C9	114.9 (4)	C22—C20—H20A	109.2
C11—C10—C9	112.8 (3)	C19—C20—H20A	109.2
C16—C10—H10A	105.6	O4—C21—O3	121.2 (3)
C11—C10—H10A	105.6	O4—C21—C20	127.4 (4)
C9—C10—H10A	105.6	O3—C21—C20	111.4 (4)
O1—C11—C10	109.3 (3)	C20—C22—H22A	109.5
O1—C11—C12	103.1 (3)	C20—C22—H22B	109.5
C10—C11—C12	118.4 (3)	H22A—C22—H22B	109.5
O1—C11—H11A	108.5	C20—C22—H22C	109.5
C10—C11—H11A	108.5	H22A—C22—H22C	109.5
C12—C11—H11A	108.5	H22B—C22—H22C	109.5
C1—C12—C13	115.3 (3)		
			
C9A—C1—C2—C3	−0.9 (5)	C9A—C1—C12—C13	122.1 (4)
C12—C1—C2—C3	179.3 (4)	C2—C1—C12—C13	−58.1 (6)
C1—C2—C3—N4	1.2 (4)	C9A—C1—C12—C11	9.3 (5)
C1—C2—C3—C18	−175.9 (4)	C2—C1—C12—C11	−170.9 (4)
C2—C3—N4—C9A	−1.1 (4)	O1—C11—C12—C1	86.1 (4)
C18—C3—N4—C9A	176.4 (3)	C10—C11—C12—C1	−34.7 (5)
C2—C3—N4—C5	−173.7 (3)	O1—C11—C12—C13	−35.8 (4)
C18—C3—N4—C5	3.8 (6)	C10—C11—C12—C13	−156.5 (4)
C9A—N4—C5—C6	−62.4 (5)	C1—C12—C13—C14	−80.9 (4)
C3—N4—C5—C6	109.1 (4)	C11—C12—C13—C14	36.6 (4)
N4—C5—C6—C7	78.0 (4)	C1—C12—C13—C15	46.5 (5)
C5—C6—C7—C8	−64.2 (5)	C11—C12—C13—C15	163.9 (4)
C6—C7—C8—C9	63.6 (5)	C11—O1—C14—O2	−178.3 (4)
C3—N4—C9A—C1	0.5 (4)	C11—O1—C14—C13	2.8 (4)
C5—N4—C9A—C1	173.3 (3)	C15—C13—C14—O2	24.3 (7)
C3—N4—C9A—C9	−175.8 (3)	C12—C13—C14—O2	155.6 (4)
C5—N4—C9A—C9	−3.0 (6)	C15—C13—C14—O1	−157.0 (3)
C2—C1—C9A—N4	0.2 (4)	C12—C13—C14—O1	−25.7 (4)
C12—C1—C9A—N4	−180.0 (4)	C11—C10—C16—C17	173.3 (4)
C2—C1—C9A—C9	176.4 (4)	C9—C10—C16—C17	−56.7 (5)
C12—C1—C9A—C9	−3.8 (6)	C21—O3—C18—C3	−141.6 (3)
N4—C9A—C9—C10	−163.9 (4)	C21—O3—C18—C19	−16.5 (4)
C1—C9A—C9—C10	20.4 (6)	C2—C3—C18—O3	117.6 (4)
N4—C9A—C9—C8	67.1 (5)	N4—C3—C18—O3	−59.2 (4)
C1—C9A—C9—C8	−108.6 (5)	C2—C3—C18—C19	−0.4 (6)
C7—C8—C9—C9A	−78.1 (4)	N4—C3—C18—C19	−177.2 (3)
C7—C8—C9—C10	157.7 (4)	O3—C18—C19—C20	23.7 (4)
C9A—C9—C10—C16	−171.9 (3)	C3—C18—C19—C20	144.0 (3)
C8—C9—C10—C16	−47.0 (5)	C18—C19—C20—C21	−22.1 (4)
C9A—C9—C10—C11	−42.5 (4)	C18—C19—C20—C22	98.3 (4)
C8—C9—C10—C11	82.3 (5)	C18—O3—C21—O4	−176.7 (4)
C14—O1—C11—C10	148.1 (3)	C18—O3—C21—C20	2.1 (5)
C14—O1—C11—C12	21.3 (4)	C22—C20—C21—O4	68.0 (6)
C16—C10—C11—O1	67.9 (4)	C19—C20—C21—O4	−168.2 (4)
C9—C10—C11—O1	−63.2 (4)	C22—C20—C21—O3	−110.7 (4)
C16—C10—C11—C12	−174.5 (3)	C19—C20—C21—O3	13.1 (5)
C9—C10—C11—C12	54.4 (5)		

### Hydrogen-bond geometry (Å, º)

**Table:**

*D*—H···*A*	*D*—H	H···*A*	*D*···*A*	*D*—H···*A*
C5—H5*A*···O2^i^	0.97	2.60	3.531 (4)	161
C5—H5*B*···O4^ii^	0.97	2.66	3.595 (3)	162
C22—H22*B*···O4^iii^	0.96	2.63	3.496 (4)	150

Symmetry codes: (i) −*x*+1, *y*−1/2, −*z*; (ii) *x*, *y*, *z*+1; (iii) *x*−1, *y*, *z*.
